# Evaluation of Divers’ Neuropsychometric Effectiveness and High-Pressure Neurological Syndrome *via* Computerized Test Battery Package and Questionnaires in Operational Setting

**DOI:** 10.3389/fphys.2019.01386

**Published:** 2019-11-08

**Authors:** Simin Berenji Ardestani, Costantino Balestra, Elena V. Bouzinova, Øyvind Loennechen, Michael Pedersen

**Affiliations:** ^1^Department of Clinical Medicine, Aarhus University, Aarhus, Denmark; ^2^Department of Circulation and Medical Imaging, Faculty of Medicine and Health Sciences, NTNU Norwegian University of Science and Technology, Trondheim, Norway; ^3^Divers Alert Network Europe - Research Division, Roseto, Italy; ^4^Environmental, Occupational, Ageing (Integrative) Physiology Laboratory, Haute Ecole Bruxelles-Brabant (HE2B), Brussels, Belgium; ^5^Department of Biomedicine, Aarhus University, Aarhus, Denmark; ^6^TechnipFMC, Stavanger, Norway

**Keywords:** high-pressure neurological syndrome, saturation diving, central nervous system, neuropsychology, arousal

## Abstract

**Introduction:** When divers are compressed to water depths deeper than 150 meter sea water (msw), symptoms of high-pressure neurological syndrome (HPNS) might appear due to rapid increase in pressure on the central nervous system during compression. The aim of this study was to first operate a new computerized tool, designed to monitor divers’ wellbeing and cognitive function, and to record the results. The second aim was to evaluate the feasibility and validity of the Physiopad software and HPNS questionnaires as a new tool for monitoring divers wellbeing in an operational setting, including sensible visualization and presentation of results.

**Methods:** The Physiopad was operated onboard Deep Arctic (TechnipFMC Diving Support Vessel). The diving work was performed between 180 and 207 msw. The data from 46 divers were collected from the HPNS questionnaires, Hand dynamometry test, Critical Flicker Fusion Frequency test (CFFF), Adaptive Visual Analog Scale (AVAS), Simple Math Process (MathProc test), Perceptual Vigilance Task (PVT), and Time Estimation Task (time-wall).

**Result:** Diver’s subjective evaluation revealed different symptoms, possibly also HPNS related, which lasted from 1 to 5 days in storage, with the common duration being 1 day. The results from Physiopad battery testing showed no signs of significant neurological alteration.

**Conclusion:** The present study showed that there was no association between subjective measurements of HPNS and neuropsychometric test results. We also confirmed the feasibility of using the computerized test battery to monitor saturation divers at work. The HPNS battery and Physiopad software could be an important tool for monitoring diver’s health in the future. This tool was not used during the Bahr Essalam project to operationally evaluate any HPNS effect on divers as data analysis was performed post-project.

## Introduction

With the development of oil and gas industry, the need for offshore commercial underwater work has accelerated. High-pressure neurological syndrome (HPNS) has been reported as a condition that may appear in diving deeper than 150 meters of seawater (msw) due to the rapid increase in pressure on central nervous system (CNS) during compression (Vaernes et al., 1988; Ozgok Kangal and Murphy-Lavoie, 2018). HPNS is characterized as a motor, sensory, and autonomic disorder as well as impaired behavioral and cognitive function (Aarli et al., 1985; Vaernes et al., 1988; Talpalar, 2007). These alternations are clinically recognized by tremor (Vaernes et al., 1983), electroencephalographic (EEG) abnormalities (Aarli et al., 1985), myoclonus (Abraini and Rostain, 1991), sleep disorders (Seo et al., 1998), nausea, headache, dizziness (Talpalar, 2007), and reduced performance in cognitive tests (Aarli et al., 1985). Normally, these symptoms will disappear during the storage phase as the body acclimatizes to the new stable pressure. The development of the symptoms was reported to be highly dependent on the diving depth and the compression rate (Ozgok Kangal and Murphy-Lavoie, 2018).

The Psychology Experiment Building Language (PEBL) battery is a free software consisting of approximately 50 different validated experimental psychology and behavioral neurology tests to evaluate cognitive function in subjects (Piper et al., 2012). Several studies have previously used selected tests of PEBL package to study cognitive function such as visual motor coordination and visual memory in divers (Germonpre et al., 2017; Lafere et al., 2019). A collection of these psychomotor tests, as well as different questionnaires, was gathered in a computerized test battery (Physiopad package, rev 2.0, Divetech, Biot, France & I-Phy, Belgium) to monitor diver cognitive function and HPNS-related symptoms. There are no existing epidemiological studies investigating the relationship between HPNS symptoms and intermediate diving depths.

The aim of this study was to evaluate cognitive function and symptoms of HPNS in operational saturation diving. We also aimed to evaluate for the first time, the feasibility and validity of using computerized package as a new tool for monitoring HPNS and diver’s health.

## Materials and Methods

### Ethics Statement

Data were collected during Bahr Essalam project operated by TechnipFMC (Aberdeen, UK) on board the “Deep Arctic” diving support vessel (DSV) in the Libyan Sea between March and September 2018. Data collection was performed at work site, all participants were employed by TechnipFMC and fully informed before recruited in the study, and no further written consents were required. The data collections were conducted according to the Declaration of Helsinki principles for ethical human experimentation (World Medical, 2013) and the database complied with the 2018 European general data protection regulations; it was fully anonymized, and the anonymization was irreversible. The institutional ethics approval was applied, and the ethics committee determined that no further approval was required since only anonymous data would be collected for this study. All divers held a valid saturation diving certificate for working as saturation divers and had passed health and fitness medical tests defined by the diving contractor. The divers also needed to pass a pre-dive medical check on board the DSV within 24 h of going into saturation.

### Data Collection

The saturation period consisted of four phases: compression, storage, decompression, and bendwatch (a period post saturation when the diver stays several hours under surveillance for arising symptoms). The diver’s compression rate was 1 msw/min upto 180 msw and 4 msw/min from 180 msw to storage depth. There was 10 min hold at 10 msw, 20 min hold at 100 msw, and 2 h hold at 180 msw. The longest diver compression from surface to 198 msw took approximately 7 h. In addition, there was a minimum stabilization time of 4 h before the divers could start the first bell run. Heliox 98/2 and Heliox 98/1 gas mixtures were used to achieve an oxygen partial pressure of 38 kPa at storage depth. The storage depth was between 170 and 198 msw depending on the work site. During storage, divers performed work slightly deeper than their storage depth (maximum 8 h bell runs), while two divers worked in the water (maximum of 6 h in-water time), the third diver remained in the diving bell as standby diver. The working depth was between 180 and 207 msw. Decompression rate was 1 m/40 min until 60 msw, 1 m/50 min until 30 msw, 1 m/60 min until 15 msw, and 1 m/80 min until surface. Six hours hold during nights was followed during the entire decompression. The longest decompression from 198 msw to surface took just under 9 days without including bendwatch. After surfacing, the divers stayed on the vessel for at least 12 h for bendwatch. All saturation protocols followed the diving contractor diving manuals, with basis in TechnipFMC document “MOS-GM-DIV-12109 Extended and Deep Diving Procedures.”

### Subjects

**Figure 1:**
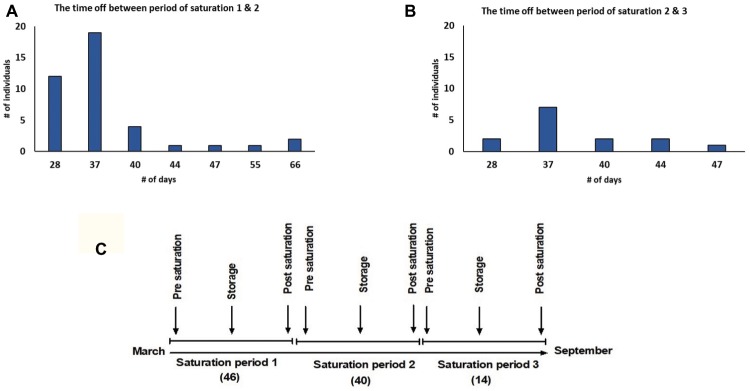
**(A)** Intermission between 1st and 2nd saturation periods; **(B)** intermission between 2nd and 3rd saturation periods; **(C)** study timeline. The study was performed between March and September 2018. Forty six divers in saturation period 1, 40 divers in saturation period 2, and 14 divers in saturation period 3 participated in the study. Data were collected during Pre-saturation, Storage, and Post-saturation periods.

There were 36 dive teams participating in this study, each team consisted of three divers. They performed a total of 500 bell runs. Divers had 1–3 saturation periods while each saturation period was 28 days for all divers except six divers who completed a period of 20 days. The time off between each saturation period was not the same for all divers, however, it was never less than 28 days; the majority of divers had 37 days off (Figures 1A,B). Data were collected from 46 divers in the first saturation period, 40 divers in the second saturation period, and 14 divers in the third saturation period (Figure 1C).

**Figure 2:**
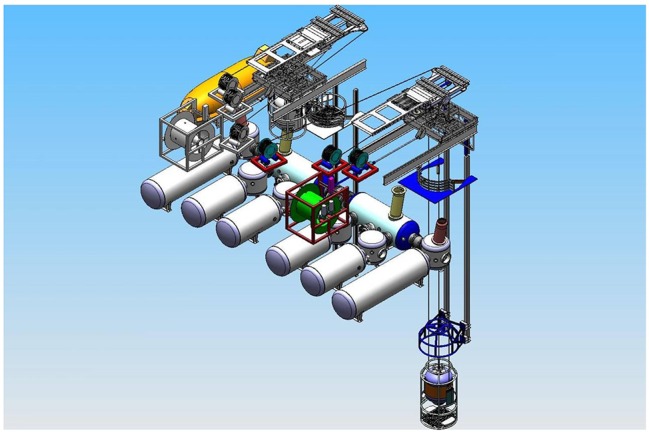
Schematic illustration of saturation system in Deep Arctic. Six living chambers were used for depth adaptation of divers.

Deep Arctic is equipped with six living chambers, of which four are three-men chambers where divers live during the work period. The two other chambers are six-men chambers, which are utilized during compression and decompression of two teams of three divers when the teams at bottom need changing out (Figure 2).

## Methods

Physiopad package is a compilation of HPNS questionnaires, Hand dynamometry test, Critical Flicker Fusion Frequency (CFFF) test, Adaptive Visual Analog Scale (AVAS), Simple Math Process (MathProc test), Perceptual Vigilance Task (PVT), and Time estimation task (time-wall). Divers were introduced to the Physiopad package during mobilization, prior to saturation. They were also provided with several instruction videos to assist them to operate the software in the situations where the instructor was unavailable (interim crew changes). Divers were instructed to perform the test every day, starting with a baseline test at surface prior to commencing saturation and as soon as they reached storage depth after compression. The testing period ended the last day (when they had returned to surface). The divers could decide themselves when to perform the testing during their shift, before or after diving. In the analysis, we used the data from Pre-saturation (baseline), Storage, and Post-saturation. The Pre- and Post-saturation session’s data were collected by using tablets (Chuwi Hi10, ShenZhen City, China) and a stationary computer was used during the saturation session.

### The High-Pressure Neurological Syndrome Questionnaire

The HPNS questionnaire consisted of questions related to known HPNS-related symptoms revealed in previous studies (Vaernes et al., 1988); visual, hearing, and temperature discomfort; nausea; vertigo; tremor; and myoclonia. Divers could grade their own discomfort from 0 (no discomfort) to 100.

### Critical Flicker Fusion Frequency Test

The Flicker device (B-Checker device, I-PHY Company, Braine l’Alleud, Belgium) was used to assess cognitive function and cortical arousal evaluating visual temporal processing. The device was described previously (Balestra et al., 2018). Correlation between cognitive performance with physiological index using CFFF in both normobaric and hyperbaric condition was demonstrated in previous studies (Hemelryck et al., 2013; Lafere et al., 2019). Subjects were instructed to hold the Flicker device at an arm’s length at eye level, to look straight at the LED light and press the button when the flickering light changes to constant fusion, read the fusion number, defined as a frequency (HZ) on the back of the device, and enter it in the related section in Physiopad software. Divers repeated this test three times to assure the accuracy of results. The Flicker device was used in the Storage phase just for the first 3 months of data collection.

### Hand Dynamometer

Hand dynamometer measures the maximal isometric hand grip strength (Mafi et al., 2012). We used two different types of hand dynamometer, three digital hand dynamometers (Camry digital, South El Monte, USA), and three manual ones (Baseline, New York, USA). The digital hand dynamometer had a range between 0 and 90 kg and the manual version between 0 and 100 kg. The hand grips were not moved around to make sure all divers use the similar hand grips for each saturation phase. Divers were instructed to hold the hand dynamometer in non-dominant hand with straight elbow and press with their maximal power, then read the number, and enter it in the related section in Physiopad software.

### Adaptive Visual Analog Scale

Computerized Adaptive Visual Analog Scale (AVAS) was used to measure (subjective) reported fatigue (Marsh-Richard et al., 2009). The AVAS uses a horizontal 100-mm line. When the divers press the Start button, the first statement appears, and divers could define their answer by moving the slider ruler with the mouse. The next statement will appear when the previous statement has been scored. The test consists of eight different statements in total (Supplementary Figure S1). During one session, the response value and time of response are registered by software. To perform statistical analysis, all data were adjusted so that the lowest response value corresponds to 0 and the highest response value corresponds to 100.

### Cognitive Function

Executive and cognitive functions of divers participating in the study were measured by The Simple Math Process test, The Perceptual Vigilance Task test, and Time Estimation test (Germonpre et al., 2017). These tests are part of PEBL software package and designed to measure working memory, vigilance, and decision making (Thomann et al., 2014; Knight and Baune, 2018).

#### Simple Math Process (MathProc Test)

In the MathProc test, the diver is given a simple mathematics test to subtract and/or add single digits and decide if the result is more or less than 5. The diver will receive the next task after answering the previous one. In total, 20 random tasks will appear each time. We measured the time took the divers to answer, number of correct/incorrect answers and total test time. The divers did not receive input regarding correctness of their answers.

#### The Perceptual Vigilance Task Test

Divers should follow the command: “click here when the red dot appears as fast as possible.” They can use mouse to click on this button as soon as they observe the red dot. The red dot appears with random delays. Software registers the delay time and the total test time. The task is repeated 20 times. Answers do not appear to the divers.

#### Time Estimation Task (Time-Wall)

In this test, the ability to estimate the speed of a moving square object when it hits the floor while it is hidden behind the wall was tested. Divers should follow the command: “click here when the square hits the floor,” they can use mouse to click on this button. The task is repeated 20 times.

### Statistical Analysis

Statistical analysis was performed using XLSTAT version 2018-1 (XLSTAT-Base, Addinsoft, France) compatible with MS Excel. All numerical data were tested for normality by using Shapiro-Wilk test and Jarque-Bera test. Kruskal-Wallis honesty test (KWH) for non-paired data and Friedman’s test for paired datasets were used to analyze non-parametric data followed by Steel-Dwass-Critchlow-Fligner procedure for multiple pairwise comparisons where appropriate. The logistic regression was used for predictive analysis and the *p* of the cumulative Chi2 distribution was reported. Mann-Whitney U Test was used to analyze the differences between symptomatic and non-symptomatic groups. Statistical differences with *p* < 0.05 were considered significant.

## Results

The demographic data for the tested groups are presented in Table 1.

**Table 1:** Demographic data.

	Total, *n* = 46
Age (years)	43 ± 7
BMI (kg/m^2^)	31 ± 4
Weight (kg)	87 ± 1
Height (cm)	181 ± 6
VO_2max_ (ml/kg/min)	47 ± 11
Saturation diving experience (years)	15 ± 8
Deepest saturation dive (m)	201 ± 5

*Data presented as mean ± SD; VO_2max_: maximal oxygen uptake*.

### High-Pressure Neurological Syndrome Questionnaires

**Figure 3:**
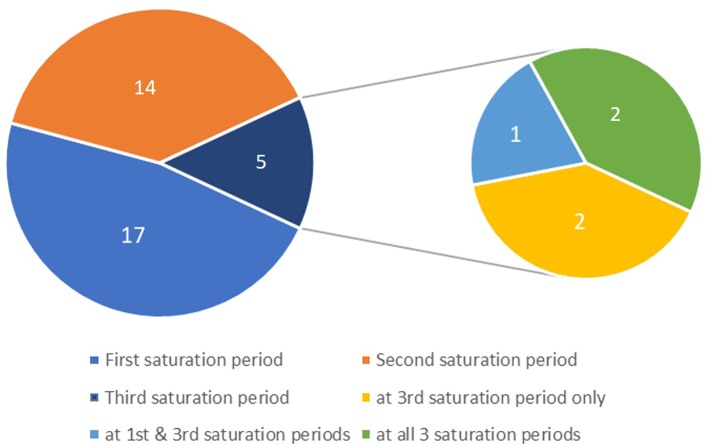
The distribution of HPNS-related symptoms during saturation periods.

**Figure 4:**
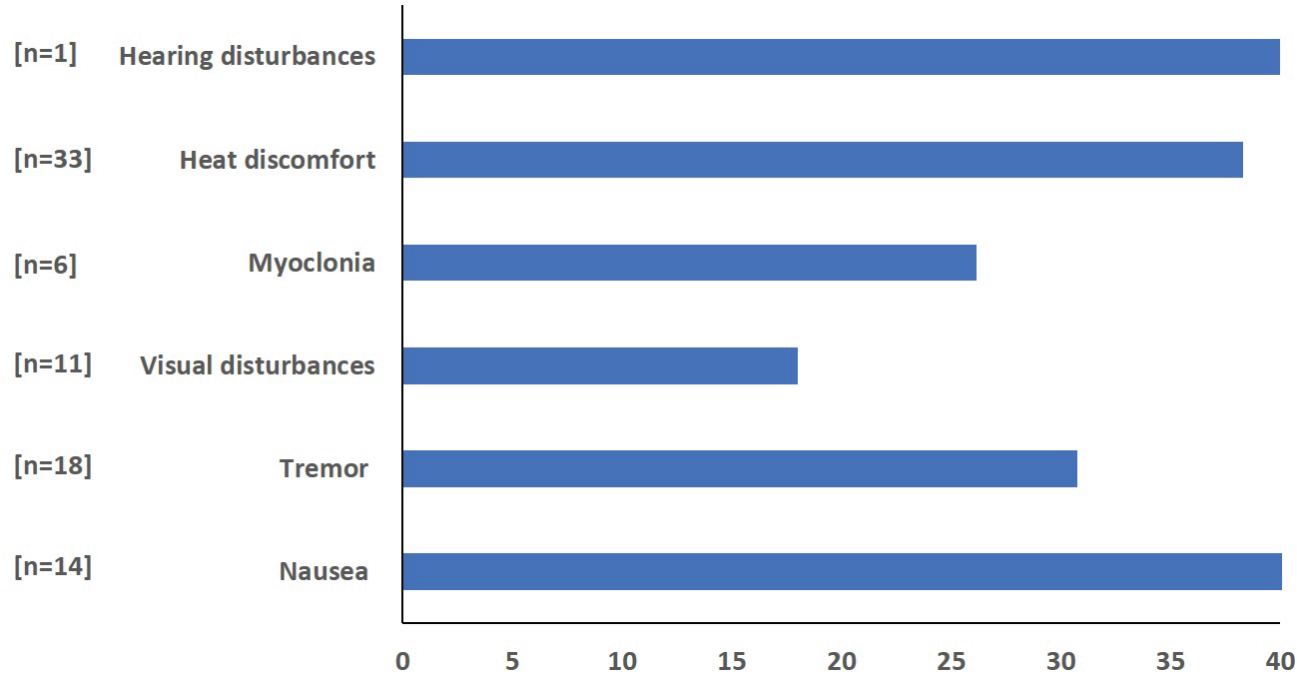
Reported discomfort during all saturation periods; 0 (no discomfort)–100.

Fifty percent (*n* = 23) of divers reported different subjective symptoms during all three saturation periods. The proportion of divers in each saturation period who reported symptoms was 37, 35, and 36% in saturation periods 1, 2, and 3, respectively. The distribution for individual reports of different symptoms is shown in Figure 3. Only two divers reported discomforts during all three saturation periods. The distribution of the reported symptoms and reported discomfort are demonstrated in Figure 4. The longest duration for the symptoms was 5 days with the common duration being 1 day. The results of logistic regression analysis showed no association between the presence of symptoms and diver’s age (*p =* 0.48), BMI (*p =* 0.83), years of experience (*p =* 0.35), or VO_2max_ level (*p =* 0.36).

### Critical Flicker Fusion Frequency Test

**Figure 5:**
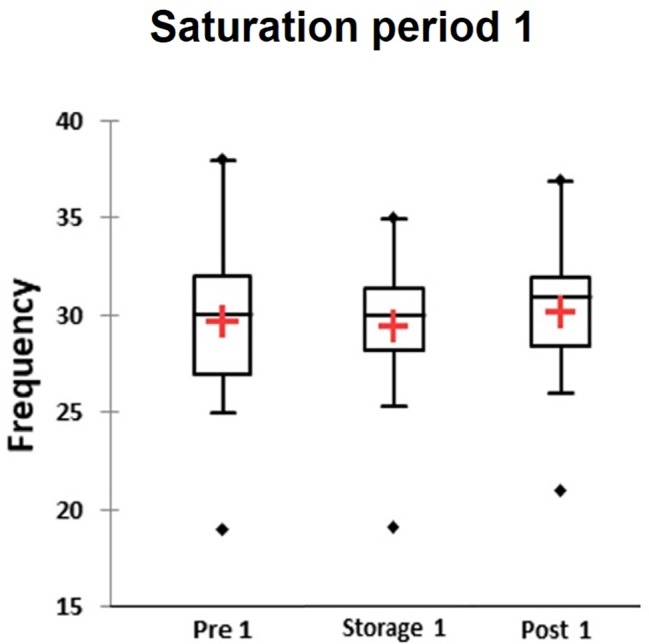
The average value from three attempts per day was calculated for each participants and results of Critical Flicker fusion frequency test presented as frequency in three different phases of the saturation period 1: Pre-saturation (Pre), Storage (Storage), and Post-saturation (Post). The red cross is mean, the horizontal line inside the boxes is median, edges of the box show inter-quartile range, and whisker indicates 95% CI.

The CFFF test was performed once per day and the value was calculated from three consecutive attempts. The CFFF data from just 36 divers during Storage phase in saturation period 1 and 24 divers in saturation period 2 were used in analysis. No differences between groups in different phases were found by KWH (Figure 5). Accordingly, multiple pairwise comparisons did not reveal any difference between phases. No difference in CFFF results between divers who reported HPNS symptoms and non-symptomatic group in saturation period 1 was observed (*U* = 174, *p =* 0.68).

### Hand Dynamometry

**Figure 6:**
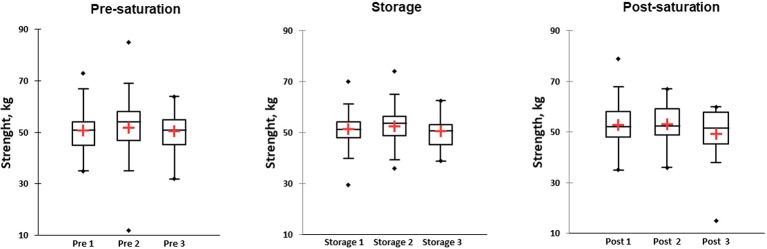
Results of hand dynamometry test represented as kilograms in three different phases of the saturation: Pre-saturation (Pre), Storage (Storage), and Post-saturation (Post) over three saturation periods (1, 2, and 3). The red cross is mean, the horizontal line inside the boxes is median, edges of the box show inter-quartile range, and whisker indicates 95% CI.

KWH was used and demonstrated no differences between groups in different phases (Figure 6, box plot for three saturation periods in Pre-saturation, Storage, and Post-saturation). Friedman’s test did not reveal any differences in groups between saturation periods (Pre-sat, Storage or Post-sat). No difference in Hand dynamometry results between divers with HPNS reported symptoms and non-symptomatic group in any saturation periods was observed (*U* = 204, *p =* 0.32).

### Adaptive Visual Analog Scales

**Figure 7:**
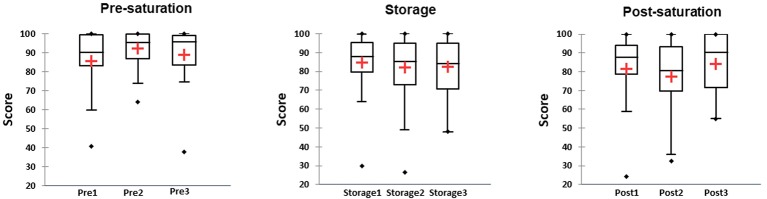
The score of the adaptive visual analogic scale (AVAS) in three different phases of the saturation: Pre-saturation (Pre), Storage (Storage), and Post-saturation (Post) for three saturation periods (1, 2, and 3). The red cross is mean, the horizontal line inside the boxes is median, edges of the box show inter-quartile range, and whisker indicates 95% CI.

Results showed increase in amount of perceived fatigue during the entire Storage phase compared to Pre-saturation. Friedman’s test did not reveal differences in test’s results in the first saturation period. In saturation period 2, there was significantly higher value in Pre-saturation in comparison to Storage and Post-saturation (Figure 7, *W*_ij_ = 1.06 *p* < 0.0001 for Pre-saturation2 vs. Storage2 and *W*_ij_ = 1.01, *p* < 0.0001 for Pre-saturation2 and Post-saturation2). Significant difference was also observed between Pre-saturation and Storage in the third saturation period (Figure 7, *W*_ij_ = 1.13 *p* = 0.006). No difference in AVAS results between divers with HPNS reported symptoms and non-symptomatic group in any saturation periods was observed (*U* = 281, *p =* 0.36).

### Simple Math Process (MathProc Test)

**Figure 8:**
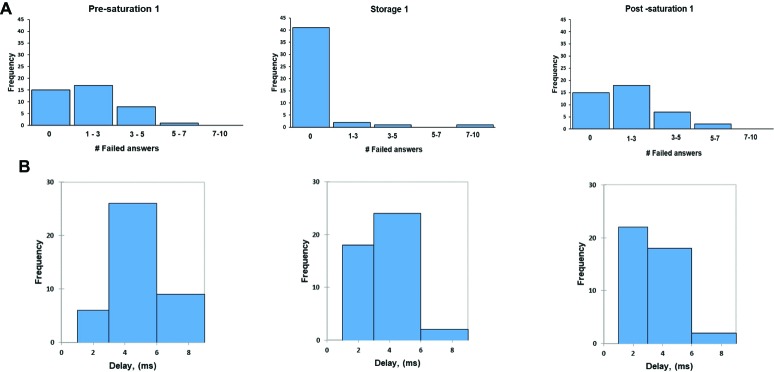
The frequency of failed answer **(A)**; the frequency of average delay **(B)** in MathProc test in three different phases in the first saturation period.

In analysis of the amount of wrong answers and delays for giving an answer, no differences between phases and saturation period were found. Divers demonstrated their ability to learn the task during the study (KWH_15.507_ = 44.100, *p* < 0.0001) and this was already achieved during the first saturation period (*W*_ij_ = −6.357, *p* < 0.0001 for Pre-saturation1 vs. Storage1 and *W*_ij_ = −5.846, *p* < 0.001 for Pre-saturation1 and Post-Saturation1). In Figure 8A, the histogram of frequency distribution is shown for all three phases and each saturation period. There were 15 participants during the first saturation period who did not make any mistakes in Pre- and Post-saturation, while all participants made at least one mistake during Storage phase. The results showed that participants replied faster with correct answer in the post-saturation phase compared to the results obtained during Storage and Pre-saturation phases (Figure 8B). No difference in wrong answers or delayed response was observed between divers with HPNS reported symptoms and non-symptomatic group in any saturation periods (*U* = 252, *p =* 0.59).

### Perceptual Vigilance Task

**Figure 9:**
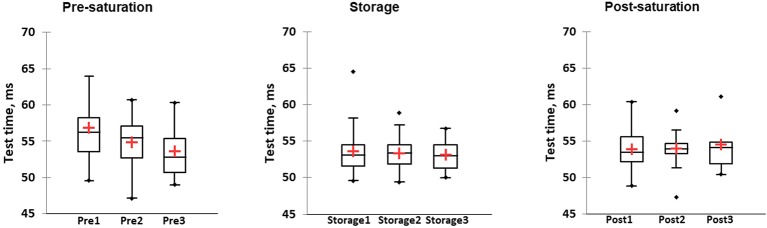
The perceptual vigilance task (PVT) test duration is presented in milliseconds in three different phases of the saturation period: Pre-saturation (Pre), Storage (Storage), and Post-saturation (Post) during three saturation periods (1, 2, and 3). The red cross is mean, the horizontal line inside the boxes is median, edges of the box show inter-quartile range, and whisker indicates 95% CI.

Based on repeated measurement using Friedman’s test, PVT test duration was significantly longer in Pre-saturation compared to Storage and Post-saturation in the first saturation period (*W*_ij_ = 0.881 *p* = 0.01 for Pre-saturation1 vs. Storage1 and *W*_ij_ = 0.583, *p* = 0.001 for Pre-saturation1 and Post-saturation1). Similar results were observed between Pre-saturation and storage in saturation period 2 (*W*_ij_ = 0.788 *p* = 0.04 for Pre-saturation2 vs. Storage2). No difference in test results between the three different saturation periods was observed (Figure 9). No difference in PVT results between divers with HPNS reported symptoms and non-symptomatic group in any saturation periods was observed (*U* = 257, *p =* 0.36).

### Time Estimation Task (Time-Wall)

**Figure 10:**
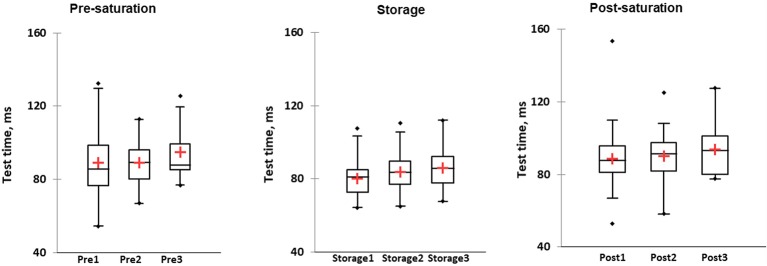
The wall-test duration is presented in milliseconds in three different phases: Pre-saturation (Pre), Storage (Storage), and Post-saturation (Post) in three saturation periods (1, 2, and 3). The red cross is mean, the horizontal line inside the boxes is median, edges of the box show inter-quartile range, and whisker indicates 95% CI.

Based on repeated measurement using Friedman’s test, time-wall test duration was significantly longer in Post-saturation compared to Storage in saturation period 1 (*W*_ij_ = 4.55 *p* = 0.034). No differences in the test results between the three different saturation periods were observed (Figure 10). No difference in time-wall duration results between divers with HPNS reported symptoms and non-symptomatic group in any saturation periods was observed (*U* = 197, *p =* 0.94).

## Discussion

In present study, we showed no differences in cognitive function or CFFF test results between divers who reported HPNS-related symptoms and who did not. This ultimately shows no association between subjective measurements and neuropsychometric test results. This study confirmed the validity and feasibility of using the computerized test battery to monitor divers’ wellbeing and psychometric efficiency at work site. In recent years, several studies have been published, evaluating cognitive performance in divers (Lafere et al., 2010; Germonpre et al., 2017); however, little has been done to look at HPNS in operational saturation diving, most references in this regard date back to the 1980s and it has received little attention in the literature (Aarli et al., 1985; Vaernes et al., 1988).

Based on previous studies, the variability of HPNS symptoms in different divers has been reported (Vaernes et al., 1988); therefore, Physiopad test battery was designed to allow both objective and subjective measurements in divers to evaluate different CNS effects by measuring the degree of vigilance, cognitive and psychomotor performance. The subjective evaluation revealed several symptoms consistent with what has previously been attributed to HPNS (Aarli et al., 1985; Vaernes et al., 1988). There were several complaints of heat discomfort, tremor, and nausea in first days of saturation. Since some of these discomforts were also experienced by divers in shallower storage depth, these symptoms are not necessarily related to HPNS. However, we should consider that 50% of the divers had reported at least one discomfort that last between 1 and 5 days in storage, while the common duration was 1 day.

Both postural and intention tremor in hand and upper limb have previously been reported as the most characteristic symptom related to HPNS (Aarli et al., 1985; Vaernes et al., 1988; Todnem et al., 1989). Postural tremor occurs when the subject is unable to maintain the limb in steady position (Anouti and Koller, 1995), while intention tremor is the “target-directed movements” and occurs at the end of the voluntary movements (McCreary et al., 2018). Several studies have described hand dynamometer as a reliable instrument to measure hand muscle and grip strength; however, the result could vary due to the type of the dynamometer, the distance from the joint, and shoulder/elbow position (Mafi et al., 2012). Moreover, the correlation between muscle strength and tremor in neurological diseases remains debated (Deuschl et al., 1998; Ozer et al., 2018). In this study, the results of Hand dynamometer testing showed no differences between phases or saturation periods. All divers received instruction about how to use the hand grip; however, most of the hand grip measurements were performed by divers without the presence of the instructor (due to operational constraints), which might lead to some inconsistency in the results. Nevertheless, these results are similar to previous studies and no difference in test results were reported (Vaernes et al., 1988), and it is unlikely that a reversible tremor causes any deficiencies in the grip strength.

The AVAS test results showed an increase in divers’ perceived fatigue during the entire Storage phase compared to Pre- and Post-saturation and these values tend to increase from first saturation toward the third saturation. Since the divers had different time off between saturation periods, this tendency might be related to individual characteristics. We could not find any association between number of days off and AVAS test results. Previously, subjective post-saturation fatigue after deep diving (300 and 350 msw) was reported while other HPNS-related symptoms were present (Aarli et al., 1985). However, diver’s post-saturation physical and mental fatigue was also reported at shallower depths (110–136 msw) (Imbert et al., 2018). Therefore, we believe that subjective fatigue is independent of HPNS or any neurological symptoms.

The results of psychometric tests in this study showed no difference between the saturation phases or saturation periods. These psychometric tests were previously shown as good assessors of cognitive function in divers (Germonpre et al., 2017). The deterioration in cognitive function due to HPNS was reported earlier in diving deeper than 200 msw (Aarli et al., 1985; Vaernes et al., 1988). The psychometric tests are criticized for a bias in leaning of repetitive tasks, while no such a bias exists with neurophysiological tests such as EEG and CFFF (Ortiz et al., 2005). The improved performance in cognitive tests, for example in the PVT test, could be due to this phenomenon or even differences in oxygen partial pressure (Imbert et al., 2018; Kiboub et al., 2018). Divers were recommended to perform the test everyday while they were in Storage, which some of them did. Also, variations in educational level might affect the results, mostly for the MathProc test.

Changes in divers’ EEG due to HPNS were reported in previous studies (Aarli et al., 1985; Vaernes et al., 1988). EEG is a reliable tool for neurophysiological tests (Smith, 2005; Berka et al., 2007) but it would not be feasible to measure it while divers are in saturation chamber and an alternative tool is needed. In recent years, several studies were published introducing Flicker as a reliable method to monitor changes in brain function and cortical arousal (Ortiz et al., 2005; Sharma et al., 2011). Flicker was used earlier to measure alertness and cognitive function in divers (Lafere et al., 2010, 2019; Germonpre et al., 2017). Decreased cognitive function was reported to be associated with a decrease in Flicker test and vice versa (Balestra et al., 2018; Lafere et al., 2019). Flicker has several limitations and the results would change due to the distance from the eyes, vision, and average brightness (Lafere et al., 2010). In this study, Flicker results were not different between saturation phases or periods, which is in line with our results of the psychomotor tests. It should be mentioned that among 18 divers who reported tremor, we had CFFF data from just two divers during Storage phase. The relative changes of the two divers that performed the CFFF test with tremor symptoms are visibly different from the general average, but the limited number of data does not allow any conclusion, further investigation is needed. This could explain why we could not see any correlation between CFFF results and reported tremor.

## Conclusion

There were no differences detected between those with subjective symptoms and those without, but no cases of HPNS were medically diagnosed, so the tool proves to be adequate in assessing divers’ efficient work capacity since all divers continue to work during the dive, no one was impeached. Nevertheless, its medical sensitivity must be ascertained after testing it upon subjects with and without medically diagnosed HPNS. We need to emphasize that the result of this study was not used during Bahr Essalam project to operationally evaluate any HPNS effect on divers as data analysis was performed post-project. We were able to collect data everyday without interfering with the diver’s job; however, there are several suggestions to improve the Physiopad software. Tremor is the most known clinical symptom of HPNS (Vaernes et al., 1988; Talpalar, 2007). In the current battery, it has been subjectively reported but there are no objective measurements of tremor. Finger oscillation test, finger-to-nose coordination, or recent smart-phone application could be different additional to the Physiopad software (Morgan et al., 1975; Vaernes et al., 1988; Garcia-Magarino et al., 2016). It is also recommended to improve the quality of the instructions and diver’s familiarization with test battery especially in using the Flicker device.

## Limitations

While, divers were instructed to perform the test as soon as they reached storage depth after compression, several divers did not follow this instruction. There was inconsistency among divers for the start of the test; therefore we decided to use the data from entire Storage phase. The authors could not give all the instruction to the divers due to the time and operational constraints to stay onboard. The Flicker devices were removed from chambers after 3 months at the start of the project due to the lithium battery restrictions. Thus, we were able to collect the Flicker data during Storage phase just for a short period. The new generation of Flicker device with AA battery and compatible with hyperbaric environment is commercially available now and could be a useful tool for future research, but we did not have access to this new Flicker device during data collection.

Moreover, there was a limitation in Phyiopad software related to given IDs. Divers mixed their IDs between stationary computers and tablets many times since the software allows them to log in with new IDs and reset the passwords. This made it difficult to keep track of the individual recordings. Having assigned IDs with predefined password would help with this situation and divers will not be able to log in with any other ID.

## Data Availability Statement

The datasets generated for this study are available on request to the corresponding author.

## Ethics Statement

Ethical review and approval were not required for the study on human participants in accordance with the local legislation and institutional requirements. Written informed consent for participation was not required for this study in accordance with the national legislation and the institutional requirements.

## Author Contributions

ØL designed the study, diving technical supervision, and report review. SB managed the data collection. SB and EB conducted the statistical analysis. SB drafted the manuscript. All the authors contributed to final correction and writing.

### Conflict of Interest

ØL is employed by TechnipFMC in Norway and CB is a founder of I-Phy in Belgium.

The remaining authors declare that the research was conducted in the absence of any commercial or financial relationships that could be construed as a potential conflict of interest.
