# Supplementary figures and images for: Evaluation of Divers’ Neuropsychometric Effectiveness and High-Pressure Neurological Syndrome via Computerized Test Battery Package and Questionnaires in Operational Setting
Source: Front Physiol. 2019 Nov 8;10:1386. doi: 10.3389/fphys.2019.01386 (PMC6856207; doi:10.3389/fphys.2019.01386)

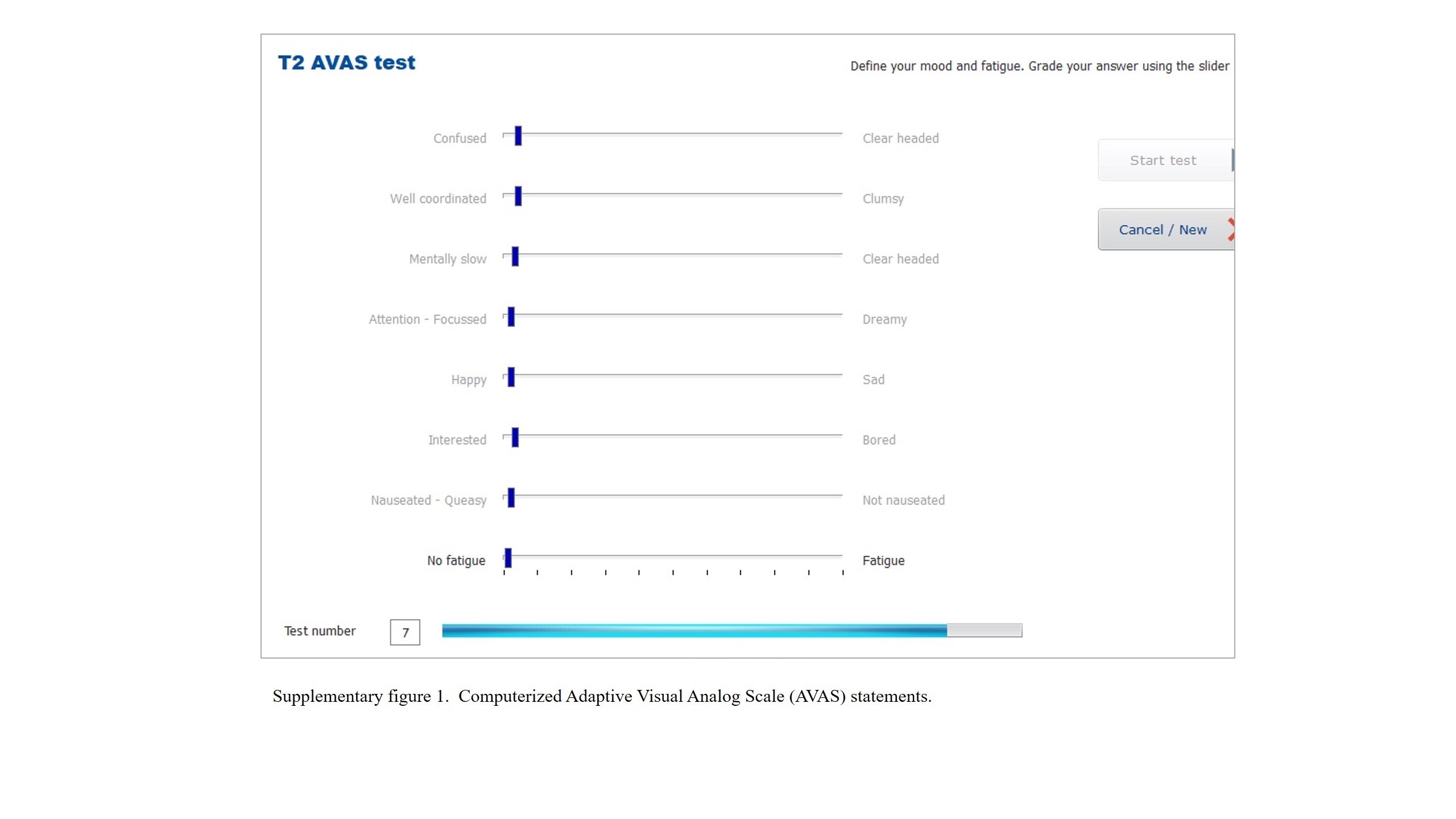

Supplement: Supplementary file 1 [file Image_1.JPEG]
